# Investigation of SNP rs2060546 Immediately Upstream to *NTN4* in a Danish Gilles de la Tourette Syndrome Cohort

**DOI:** 10.3389/fnins.2016.00531

**Published:** 2016-11-22

**Authors:** Shanmukha S. Padmanabhuni, Rayan Houssari, Ann-Louise Esserlind, Jes Olesen, Thomas M. Werge, Thomas F. Hansen, Birgitte Bertelsen, Fotis Tsetsos, Peristera Paschou, Zeynep Tümer

**Affiliations:** ^1^Department of Molecular Biology and Genetics, Democritus University of ThraceAlexandroupoli, Greece; ^2^Applied Human Molecular Genetics, Kennedy Center, Department of Clinical Genetics, Copenhagen University HospitalRigshospitalet, Glostrup, Denmark; ^3^Danish Headache Center and Department of Neurology, Faculty of Health Sciences, University of Copenhagen, Glostrup HospitalGlostrup, Denmark; ^4^Institute of Biological Psychiatry, Mental Health Center Sct. Hans, Copenhagen University HospitalRoskilde, Denmark; ^5^Department of Biological Sciences, Purdue UniversityWest Lafayette, IN, USA

**Keywords:** axon guidance, Gilles de la Tourette syndrome, GTS, Netrin-4, *NTN4*, single nucleotide polymorphism, SNP

## Abstract

Gilles de la Tourette syndrome (GTS) is a neuropsychiatric disorder characterized by multiple motor and vocal tics. GTS is a complex disorder, with environmental factors and several genes involved. Although variations within a few genes such as *AADAC, NRXN1, SLITRK1, HDC*, and *IMMP2L* have been tentatively associated with GTS (in a small number of patients), the causative genes underlying GTS pathophysiology remain unknown. In a previous genome-wide association study (GWAS) a single nucleotide polymorphism (SNP, rs2060546) near the Netrin-4 (*NTN4* - MIM 610401) gene was shown to be associated with GTS [odds ratio (OR) = 1.7; *p*-value = 5.8 × 10-7] thus warranting further investigations. As *NTN4* is one of the axon guidance molecules expressed in the central nervous system and it interacts with the encoded proteins of *SLIT* and *WNT* genes guiding the growth cone toward its target, it is an attractive candidate susceptibility gene for GTS. In this study we attempted to replicate the association of rs2060546 with GTS by genotyping a Danish cohort of 240 GTS patients and 1006 healthy controls. Our results did not reveal an association (OR = 1.363; *p*-value = 0.3329) in the Danish cohort alone, which may be due to the small sample size. However, a meta-analysis including the present cohort and a total of 1316 GTS patients and 5023 controls from the GTS GWAS Replication Initiative (GGRI) and the first GTS-GWAS yielded a significant signal (OR = 3.74; *p*-value = 0.00018) and same direction of effect in the three cohorts. Thus, our study strengthens the evidence of the possible involvement of *NTN4* in GTS etiology, suggesting that further studies in even larger samples and functional studies are warranted to investigate the role of this region in GTS pathogenesis.

## Introduction

Gilles de la Tourette syndrome (GTS) is a complex juvenile-onset neuro-developmental disorder characterized by the occurrence of multiple motor and vocal tics (Nag et al., 2013). Recent epidemiological studies estimated the worldwide prevalence of GTS to be approximately 1% with a male:female ratio of 4:1 (Robertson et al., 2009). GTS is often associated with co-morbidities such as attention-deficit hyperactivity disorder and obsessive-compulsive disorder (Dietrich et al., 2014). Additional co-occurring conditions are behavioral and emotional difficulties (hyperactivity, anxiety, and depression), sleeping disturbances, intellectual disabilities and autism spectrum disorder (Singer and Rosenberg, 1988; Wood et al., 2003). Studies so far conducted on GTS showed that there is a complex interplay of environmental and genetic factors, confirming the notion that GTS is a highly complex disorder (Davis et al., 2013; Dietrich et al., 2014).

To identify the causative genes and the biological pathways involved in GTS several approaches have been pursued, including candidate gene studies, family studies using linkage analysis, analysis of chromosomal abnormalities including copy number variants (CNV) and hypothesis-free genome-wide association studies (GWAS) (Paschou, 2013). For instance, chromosomal abnormalities have proven useful for identifying new candidate genes in GTS affected patients (Bertelsen et al., 2013). Chromosomal abnormalities have probed new candidate regions containing susceptible genes such as Slit- and Ntrk-Like Family, Member 1 (*SLITRK1*—MIM 609678), Neuroligin 4 (*NLGN4*—MIM 300427) and Contactin-Associated Protein-Like2 (*CNTNAP2*—MIM 604569) (Verkerk et al., 2003; Abelson et al., 2005; Lawson-Yuen et al., 2008; Patel et al., 2011). Specific CNV analyses have resulted in the identification of several other candidate genes, including Arylacetamide Deacetylase (*AADAC*—MIM 600338), Collagen, Type VIII, Alpha-1 (*COL8A1—*MIM 120251), Neurexin I (*NRXN1—*MIM 00565), Catenin Alpha-3 (*CTNNA3*—MIM 607667) and Inner Mitochondrial Membrane Peptidase, Subunit 2, (*IMMP2L—MIM* 605977) (Sundaram et al., 2010; Nag et al., 2013; Bertelsen et al., 2014, 2015). The first large GWAS for GTS did not identify any genome-wide significance SNP (Scharf et al., 2013). A multinational consortium, GTS GWAS Replication Initiative (GGRI), followed up on the results of the initial GWAS in an independent cohort of 636 cases and 641 controls, showing an association between GTS and rs2060546 (OR = 2.41; *p*-value = 5.8 × 10^−7^) on chromosome 12q23.1, ~32 kb upstream to the transcriptional start site of Netrin-4 (*NTN4)*; a gene encoding an axon guidance protein expressed in the central nervous system (Paschou et al., 2014). As *NTN4* is an attractive candidate susceptibility gene, we investigated the association of rs2060546 with GTS in a Danish cohort of 240 GTS patients and 1006 healthy controls.

## Materials and methods

### Samples

We investigated a Danish cohort and two previously published datasets (GGRI and GTS-GWAS) (Table 1). The first cohort (*n* = 1314) includes 261 deeply phenotyped GTS patients and 1053 healthy controls from Denmark. Danish ancestry was investigated using the genotype information. The second cohort from GGRI includes 1277 individuals (636 GTS cases and 641 healthy controls) from different European populations (Paschou et al., 2014). The third cohort (GTS-GWAS) includes 6249 individuals (1285 GTS cases and 4964 controls) of European ancestry (Scharf et al., 2013).

**Table 1:** **Summary of the three different cohorts included in the meta-analysis**.

**Cohort**	**Cases**				**Controls**			
	**Male**	**Female**	**Unknown**	**Total**	**Male**	**Female**	**Unknown**	**Total**
Danish	208	53	0	261	649	403	1	1053
GGRI	498	127	11	636	346	284	11	641
GTS-GWAS	1012	273	0	1285	1931	3033	0	4964

### Genotyping

261 GTS cases from Denmark were genotyped on the Affymetrix CytoScanHD array (Affymetrix, Santa Clara, CA) covering around 750,000 single nucleotide polymorphisms (SNPs) and ~1.9 million single-locus copy number (CN) markers. 1053 healthy controls were genotyped on Illumina HumanOmniExpress arrays—18 on HumanOmniExpress12v1 and 1034 on HumanOmniExpress12v1-1 covering around 700,000 SNPs each. SNP calling and pre-processing of the raw data for Illumina controls were carried out using Illumina Genomestudio® software. For GTS cases genotyped on Affymetrix CytoScanHD SNP calling a pre-processing was carried out using default parameters for method apt-copynumber-cyto from Affymetrix Power Tools (APT, version 1.16) software.

### Quality control

**Figure 1:**
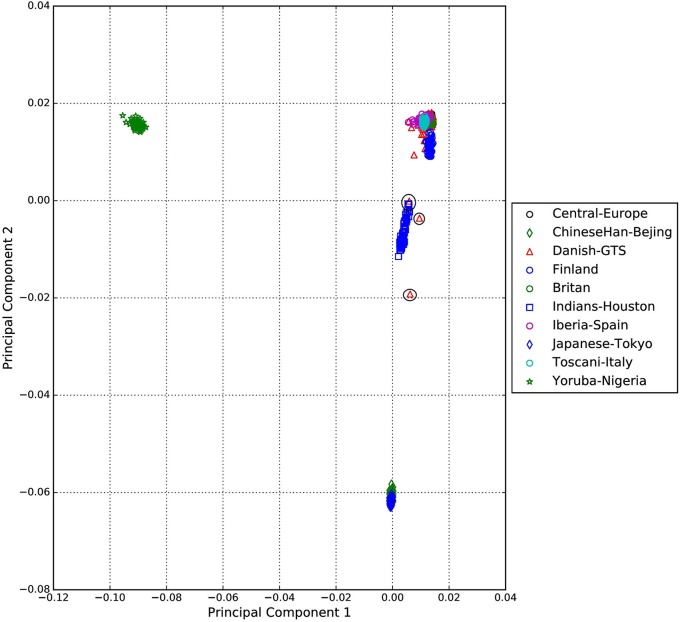
**Two dimensional PCA plot of Danish GTS cases with selected populations from 1000genomes cohort**.

**Figure 2:**
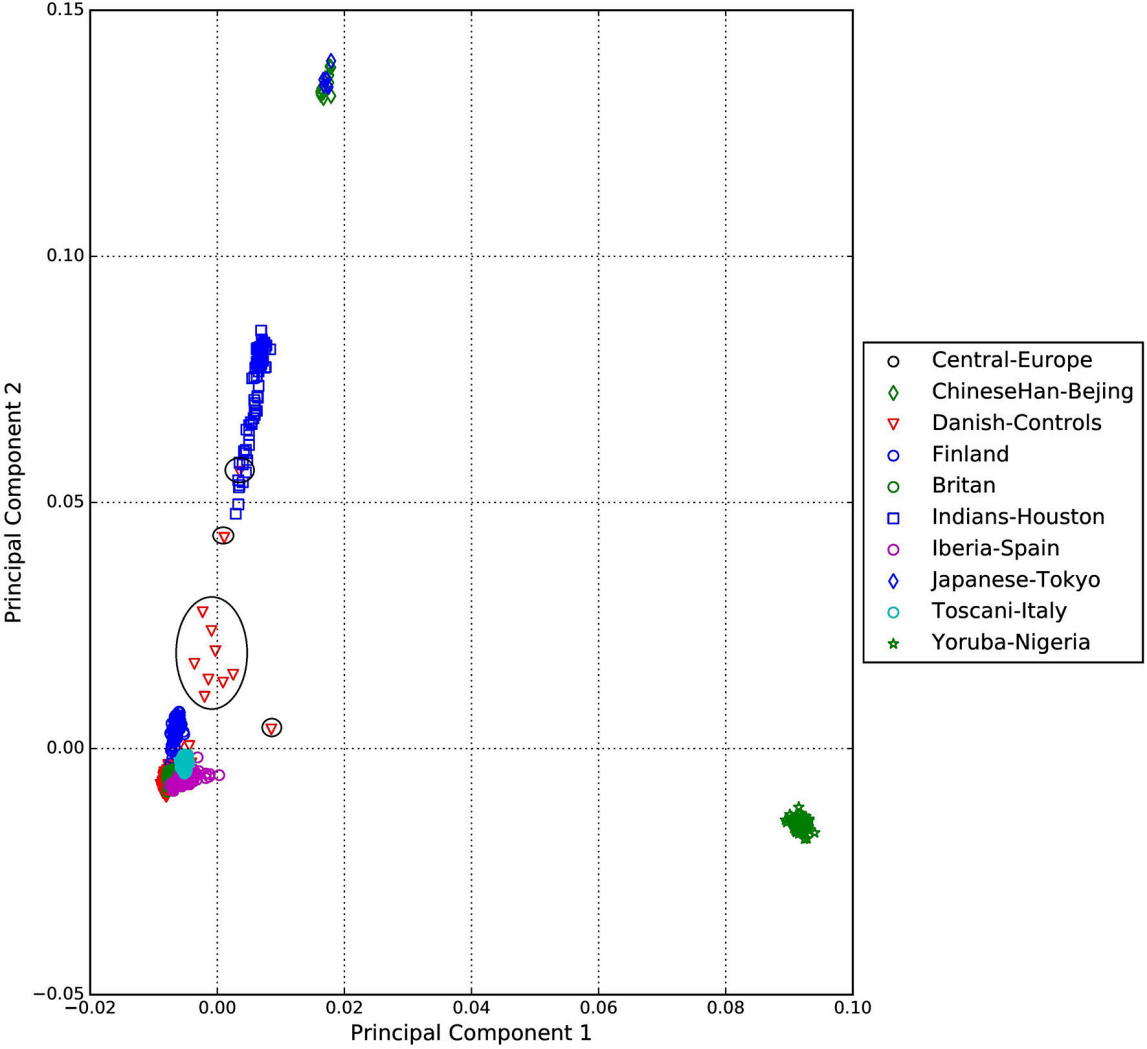
**Two dimensional PCA plots of Danish controls and selected populations of 1000genomes cohort**.

**Figure 3:**
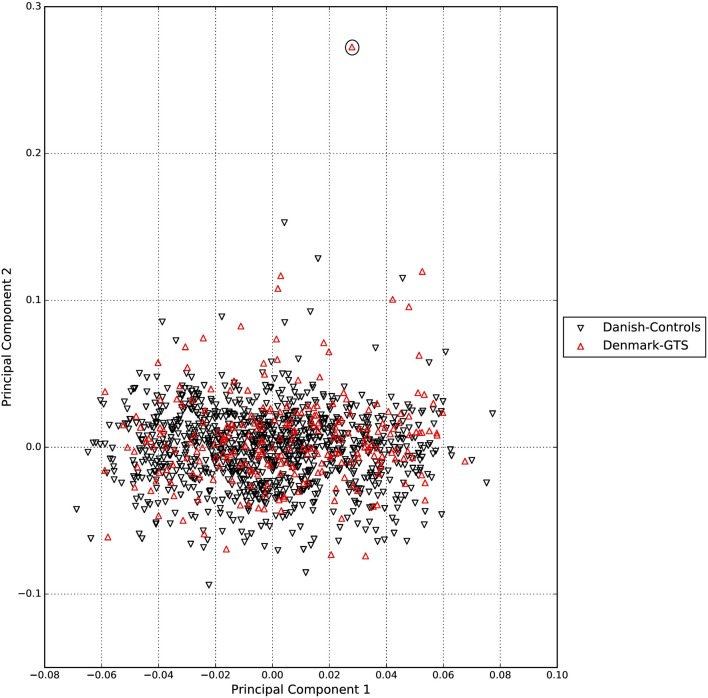
**Two dimensional PCA plot of Danish GTS cases and controls to check for population outliers**.

A standard GWAS quality control measure (Scharf et al., 2013) was applied on both cases and controls from the Danish cohort to filter for population outliers and samples with low call rate; and to remove systematic bias using the software PLINK (Purcell et al., 2007). Quality control at SNP level includes removal of monomorphic SNPs, SNPs with genotyping rate <98%, SNPs with no information about chromosome location, SNPs with absolute minor allele frequency difference > 0.15, SNPs that fail Hardy-Weinberg Equilibrium and SNPs with AT/CG alleles. To check the quality of the Danish GTS cases metric values for CytoScanHD array recommended by the manufacture was used in the pre-processing step. Sample call rate calculated using PLINK (Purcell et al., 2007) was used to filter out samples that had more than 2% of SNPs missing. F statistic was calculated using SNPs from the X-chromosome. *F* <0.25 was assigned to female and *F* > 0.75 was assigned to male. Samples with an *F*-value between 0.25 and 0.75 were discarded due to sex ambiguity. Estimates of pairwise Identity-by-descent from PLINK (Purcell et al., 2007) were used to remove one of the samples from each pair which pass the relatedness metrics either pi-hat > 0.1 or Z1 > 0.2. Samples with high rates of heterozygosity *F*_het_ > ± 0.05 are more likely to be result of contamination and were removed from the analysis. Principal Component Analysis (PCA) was applied using EIGENSTRAT (Price et al., 2006) and remaining samples were merged with the 1000 genomes cohort to remove European cluster and population outliers. Out of 261 GTS cases, four samples were removed as they did not lie inside the European cluster in Figure 1. Eleven healthy controls lied outside of the European cluster in Figure 2 and were removed from the association analysis. After removing the European outliers, Danish GTS cases and healthy controls were merged for 126,821 common SNPs. The PCA plot in Figure 3 was made using a LD-pruned set of ~90,000 SNPs to identify Danish outliers. One GTS case was removed as population outlier seen in Figure 3 which left in total 240 Danish GTS cases and 1006 healthy controls.

### Association analysis

Standard case control association analysis using PLINK (Purcell et al., 2007) was performed using χ^2^ test comparing SNP frequency between cases and controls.

### Meta-analysis

Metal (Willer et al., 2010) was used to perform sample weighted meta-analysis of association results of the SNP rs2060546 from all three cohorts. Association results for the GGRI and GTS-GWAS cohorts were taken from the original studies (Scharf et al., 2013; Paschou et al., 2014).

## Results

We attempted to replicate the association of SNP rs2060546 with GTS by investigating quality control passed genotyped data of 240 GTS patients and 1006 healthy controls from Denmark (Table 2). We did not find the SNP rs2060546 to be significantly associated in the Danish cohort (OR = 1.363, 95% CI 0.7188-2.675; *p*-value = 0.3329) (Table 3). This might be attributed to the small size of the cohort and we proceeded to perform a meta-analysis with previously published data (Paschou et al., 2014). However, meta-analysis of the Danish, the GGRI and the first GTS-GWAS cohorts, showed significant association to the studied SNP (OR = 3.74; *p*-value = 0.00018) and the same direction of effect in all three cohorts (Table 3). Thus, the results supported the involvement of this particular chromosomal region in GTS etiology, and potentially *NTN4*.

**Table 2:** **Quality control steps at sample level with number of samples failed at each step**.

**Quality Control (QC) Step**	**Danish GTS Affymetrix CytoScanHD**	**Danish Controls Illumina OmniExpress**
Samples before QC	261	1053
Pre-processing SNP quality	10	0
Sample Call Rate <98%	0	2
Sex ambiguous samples	0	0
Low level related samples	1	26
Abnormal heterozygosity	5	8
European Outliers	4	11
Danish Outliers	1	0
Final Samples after QC	240	1006

**Table 3:** **The association results for *NTN4*—rs2060546 SNP in the three different cohorts**.

**Cohort**	**Minor allele**	**Mino allele frequency in GTS cases**	**Minor allele frequency in controls**	***p*-value**	**Odds ratio (OR)**
Danish	A	0.03099	0.02293	0.3329	1.363
GGRI	A	0.04844	0.02131	0.00033	2.41
GTS-GWAS	A	0.03834	0.02746	0.02	1.44

## Discussion

Involvement of NTN4 in neurodevelopment makes it an attractive candidate protein as a contributing factor to GTS pathology. During the development of the nervous system, numerous dynamic guidance cues direct the trajectory of the migrating developed axon toward its suitable target (Killeen and Sybingco, 2008). Netrins are axon guidance cues which are composed of six members: Netrin 1-4 Netrin-related molecules, Netrin-G1 and G2 (Davis et al., 2013). Netrin-4 functions as guidance cue for axonal growth, neurite elongation, neuronal remodeling and plasticity (Zhang et al., 2004). The neuronal growth cones sense Netrin-4 as either attractant or repellent cues, depending on different receptors expressed on their surface or differences in the cellular signal transduction machinery (Koch et al., 2000; Qin et al., 2007). Netrin-4 might mediate axon outgrowth by attracting actions through deleted in colorectal carcinoma (DCC) or neogenin (Neo 1) receptors and repulsive effects through unc-5 homolog (UNC5A) receptors (Qin et al., 2007; Schubert et al., 2009). Previous imaging studies showed that changes in the thalamus and cerebral cortex volumes play a significant role in regulating the severity of tic symptoms implicated in GTS cases (Rueda et al., 2005; Rickards et al., 2008). Notably, expression of the *NTN4* gene was detected in human brain regions such as cerebellum, thalamus, cerebral cortex and olfactory bulb, thus, supporting the role of this gene in GTS pathogenesis (Zhang et al., 2004; Amat et al., 2006; Lee et al., 2006).

In this study, we could not identify any association between rs2060546 SNP and GTS in a relatively small Danish cohort alone, however, meta-analyses with the GGRI and GTS-GWAS cohorts provides further support for the possible association of this SNP with GTS in European populations. The close localization of rs2060546 to *NTN4* and the abundant expression of Netrin-4 in different brain regions, associated with GTS pathogenesis, rendered further support to the hypothesis that *NTN4* might be a new candidate gene for GTS.

## Author contributions

SP, performed data analysis, interpretation, and participated in manuscript writing. ZT and PP supervised the study, interpretation of the results, and participated in manuscript writing. RH participated in biological relevance of result and manuscript writing. FT participated in data analysis and manuscript writing. AE, JO, TW, TH, ZT, BB provided the data and participated in manuscript writing. All authors read and approved the final version to be published and agreed to be accountable for all aspects of the work.

## Funding

This project was supported by FP7-People-2012-ITN, project: TS-EUROTRAIN, grant number 316978; and Lundbeck Foundation, grant number R24-A2419 and R100-A9332.

### Conflict of interest statement

The authors declare that the research was conducted in the absence of any commercial or financial relationships that could be construed as a potential conflict of interest.
